# Federated horizontally partitioned principal component analysis for biomedical applications

**DOI:** 10.1093/bioadv/vbac026

**Published:** 2022-04-26

**Authors:** Anne Hartebrodt, Richard Röttger

**Affiliations:** Department of Mathematics and Computer Science, University of Southern Denmark, Odense 5230, Denmark

## Abstract

**Motivation:**

Federated learning enables privacy-preserving machine learning in the medical domain because the sensitive patient data remain with the owner and only parameters are exchanged between the data holders. The federated scenario introduces specific challenges related to the decentralized nature of the data, such as batch effects and differences in study population between the sites. Here, we investigate the challenges of moving classical analysis methods to the federated domain, specifically principal component analysis (PCA), a versatile and widely used tool, often serving as an initial step in machine learning and visualization workflows. We provide implementations of different federated PCA algorithms and evaluate them regarding their accuracy for high-dimensional biological data using realistic sample distributions over multiple data sites, and their ability to preserve downstream analyses.

**Results:**

Federated subspace iteration converges to the centralized solution even for unfavorable data distributions, while approximate methods introduce error. Larger sample sizes at the study sites lead to better accuracy of the approximate methods. Approximate methods may be sufficient for coarse data visualization, but are vulnerable to outliers and batch effects. Before the analysis, the PCA algorithm, as well as the number of eigenvectors should be considered carefully to avoid unnecessary communication overhead.

**Availability and implementation:**

Simulation code and notebooks for federated PCA can be found at https://gitlab.com/roettgerlab/federatedPCA; the code for the federated app is available at https://github.com/AnneHartebrodt/fc-federated-pca

**Supplementary information:**

Supplementary data are available at *Bioinformatics Advances* online.

## 1 Introduction

**Fig. 1.**
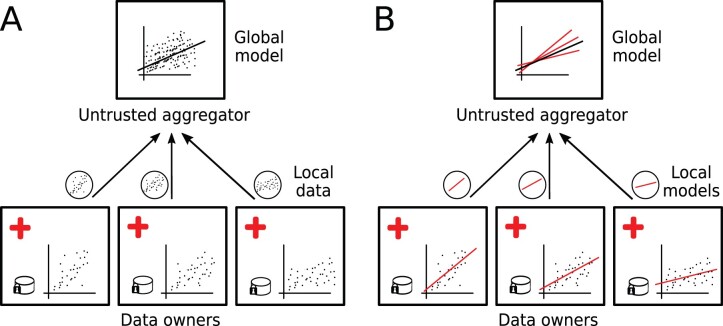
Comparison of cloud learning and FL. In cloud learning (**A**), the data are consolidated at a central server which computes the global model. In FL (**B**), the different sites (e.g. hospitals) calculate a local model on their private data and send only the model parameters to an untrusted aggregator. The global model is computed and can be sent back to the local sites

Federated learning (FL) has recently gained attention in the machine learning (ML) community as a privacy-preserving alternative to centralized computation. Contrary to classical ML, where the data are consolidated into a single machine or cloud, the data stay with the owner during the entire learning process and only model parameters are exchanged between the participants. The concept has potential applications in domains where the volume of data is too large to be stored at a single location, and in domains where the owners have concerns about losing agency over their data or are not allowed to share their data. This is especially important in the medical domain, where, due to patient confidentiality, doctors and hospitals are rightfully unable or unwilling to share their data with a third party. Outside of academic applications or in hybrid settings, FL can enable (industry) partners who are unwilling to disclose their raw data, but willing to join an analysis, to contribute to studies. These scenarios are cases of cross-silo FL where larger chunks of data are stored in ‘data silos’. Figure 1 shows a comparison between traditional, cloud based, ML, and cross-silo federated learning. Another type of FL is cross-device FL popular in particular for mobile applications, where each participant has only access to their own data (e.g. on their phone). A complementary approach for private data analysis currently discussed is the generation of synthetic data with the same properties as the raw data. This approach is a valid option if sufficiently trustworthy generators can be created (Beaulieu-Jones *et al.*, 2019; Gootjes-Dreesbach *et al.*, 2020). The advantage of FL is that it is a generic approach while synthetic data could suffer the biases of the training data and crucial, subtle information can potentially be lost in the generation process.

In the last decade, high-throughput techniques have been routinely used to generate vast amounts of biomedical data (Martin-Sanchez and Verspoor, 2014). Nevertheless, to this day, studies are commonly reporting a lack of data as a main limitation of their study, resulting in insufficiently validated, and potentially confounded, or unstable predictors (Kourou *et al.*, 2015). In the area of rare diseases for example, an investigation of the causes for trial termination showed that 30% of noncompleted clinical trials were terminated due to insufficient patient accrual (Rees *et al.*, 2019). Furthermore, many diagnostic tools are biased toward the predominant demographic at the site of the study, leading to potentially inapplicable results in other demographic groups posing an ethical problem (Wiens *et al.*, 2019). Another prominent example of the restricted data sharing is genome-wide association studies (GWAS) that suffer from a massive bias toward populations of European ancestry (Sirugo *et al.*, 2019) and very small cohorts otherwise. This problem arises because the data generated in a research facility or hospital may only leave this institution under restricted conditions (Ben van Veen, 2018), so researchers fall back to well-known cohorts. In the future, the data may even be more decentralized in ‘micro biobanks’, where each citizen has full access to their own data (Wijmenga and Zhernakova, 2018). To address this challenge, FL has been brought forward as a solution to work with sensitive medical data without breaching patient privacy.

A popular method for the analysis of biomedical data is principal component analysis (PCA). It is a dimensionality reduction technique frequently used for high-throughput sequencing count data, including bulk and single-cell transcriptome data (Theis, 2019). Several algorithms have been proposed for PCA in a federated setting. However, these algorithms were mainly evaluated using ‘standard’ test datasets and rarely with biomedical applications in mind. For instance, where the popular MNIST (LeCun *et al.*, 2005) dataset comprises 60 000 samples with 784 pixels (dimensions), bulk transcriptome data usually only have a few hundred samples but measurements for about 20 000 coding genes. The dimensionalities of these datasets are fundamentally different with *large n, moderate d* in the classical case and *small n, large d* in the biomedical scenario.

To enable the routine use of federated PCA in biomedicine, the existing algorithms must be evaluated for their suitability to analyze medical data. A major concern is the accuracy of the methods, given that the outcome of the studies will be included into medical decision-making. Here, we will investigate the suitability of various approaches to federated PCA with varying, but realistic sample distributions using data from The Cancer Genome Atlas (TCGA). TCGA is a large-scale project with multiple participating research centers (Weinstein *et al.*, 2013), which profiled various cancer types using different *OMICS* technologies, including genome and transcriptome sequencing. The decentralized nature of the TCGA sampling process makes it suitable to study the feasibility of FL using real medical data: At the tissue source site (TSS), a sample was collected and the RNA was extracted. The processed sample was then shipped to a central sequencing center, sequenced and processed according to a standardized protocol. In this setup, the sequencing was done at a central facility, which is a slight deviation from a truly federated sample acquisition process where every TSS would perform the sequencing itself. However, it is a realistic example with respect to the number and distribution of participants, potential batch effects through different population demographics per sample site and batch effects due to the sample preparation as it would occur in a truly federated analysis. Additionally, we use a multicentric Psoriasis dataset (Federico *et al.*, 2020) to show how the methods work in the presence of strong batch effects. The studies were conducted independently, but underwent a common computational preprocessing in order to allow for joint analyses.

Computational biology is a notoriously heterogeneous research field with many practitioners not having a profound background in computer science and programming. Here, we provide simulation code and a federated app that allows users to run federated PCA. Based on our considerations, we support the users with our derived guidelines to choose the appropriate algorithm for their purpose.

Overall, our contributions are:

- A comprehensive overview of different federated PCA algorithms, including simulation code and a federated app.
- Performance comparisons of those approaches, including communication overhead and different accuracy metrics.
- Evaluation of the algorithms using realistic data partitions derived from the sample distributions from TCGA.
- A practical illustration of the application of federated PCA using RNASeq data, in particular to highlight the feasibility and potential problems of federated biomedical studies.
- Guidelines for the selection of the best algorithmic approach.

The remainder of this article is structured as follows. In Section 2, we discuss the relevant data, algorithms and test setup. In Section 3, we describe our theoretical and practical findings. Section 4 puts the results into perspective and provides guidelines for the interested reader, and Section 5 concludes the work.

## 2 Methods

### 2.1 Distributed data model

The distributed setting for the remainder of the paper is as follows: The data **A** is stored in *s* distinct subsets $A=A_{1}\cup \ldots \cup A_{s}$ at *s* different sites (e.g. hospitals) and constitutes a total of *n* patients with *d* dimensions. Rows correspond to patients, columns correspond to variables. Every site has a different subset of *n_s_* patients but the full set of observed variables. Using terminology established by Ángel Rodríguez *et al.* (2017) and Wu *et al.* (2018), we speak of distributed rows or horizontal partitioning of the data. Due to privacy constraints, the sites are only allowed to exchange aggregated parameters. We are assuming a client-server/star-like architecture (Imtiaz and Sarwate, 2018; Steed and Oliveira, 2010), where sites communicate with a central server that performs the aggregation step. Peer-to-peer architectures, such as proposed in the concept of swarm learning and the personal health train (Beyan *et al.*, 2020; Warnat-Herresthal *et al*., 2021) could be used at the cost of additional communication steps and conceptually more involved protocols. The datasets at the distant sites will be called *local datasets* and the parameters or models learned using these data will be called *local parameters* or *local models*, while the final aggregated model will be called *global model* and considered optimal when it equals the result of the conventional model, the *centralized model*, calculated on all data.

### 2.2 High-dimensional biomedical data

The dimensionality of *OMICS* data can be quite unfavorable, with a high number of features compared to the number of available samples (*d *>* n*). Although the trend toward more granular (e.g. single cell) analyses alleviates this problem, and PCA is a method applied to all types of data, there are applications where the number of samples remains low. Therefore, it is interesting to evaluate, how well-federated methods perform on data with a high number of features compared to the number of samples. This setting is rarely considered in typical test scenarios for new algorithms, where usually the sample size of the test data exceeds the number of dimensions. For this study, we selected all publicly available gene expression studies on TCGA in form of the processed count tables downloaded from the web repository (https://www.cancer.gov/tcga). These contain fragments per kilobase of transcript per million fragments mapped (FPKM) normalized counts, according to the unified TCGA pipeline. We scaled and normalized the data to unit variance, but did no further processing. We chose to divide the data according to the cancer type annotated in TCGA. We narrowed down the data selection to studies containing more than 300 individuals. We split the data into subsets according to the TSS. The sample distribution over different sites varies greatly between the studies. Most of the studies have skewed sample distributions with one site contributing considerably more samples than others. Please refer to Supplementary Figure S1 and Table 1 for an overview of the number of samples and the number of TSS per study after filtering. We want to emphasize that this setup is distinctively different than the usual test setup of federated algorithms, where large datasets are split into a few equally sized chunks with iid data distribution with respect to the classes.

**Table 1.** Summary of number of samples and number of sites per cancer type

Dataset	No. of samples	No. of sites
Kidney	887	24
Thyroid gland	504	11
Liver and intrahepatic bile ducts	404	8
Bladder	408	14
Ovary	377	9
Brain	679	20
Prostate gland	495	14
Corpus uteri	547	12
Breast	1093	19
Cervix uteri	304	8
Colon	458	12
Bronchus and lung	1017	34
Stomach	386	9
Skin	468	11

### 2.3 Principal component analysis

PCA is used to calculate a low-dimensional approximation of the data (Ian, 2002). Intuitively, the data are projected into a lower-dimensional representation using the directions which maximize the variance. Let the global data be given as a matrix $A\in R^{n\times d}$. **A** is centered and scaled to unit variance. The PCA is the decomposition of the covariance matrix $M=\frac{1}{n}A^{⊤}A$ into $M=V\Sigma V^{⊤}$. Σ is a diagonal matrix containing the non-negative eigenvalues *σ_i_* in nonincreasing order. **V** is a matrix containing the eigenvectors $v_{i}$ corresponding to the eigenvalues *σ_i_* with $v_{i}$ column vectors. Note: As we are solving the eigendecomposition of a *n *×* d* matrix where d≫n the maximal number of nonzero eigenvalues is *n−*1. The top *k*-eigenvalues and corresponding eigenvectors are called a *k*-subspace and denoted as $\left(V^{k},\Sigma^{k}\right)$.

### 2.4 Federated PCA for horizontally partitioned data

In the federated case, the data are distributed over *s* sites with *n_s_* samples, such that $n=\sum_{i=1}^{s}n_{i}$. The goal of the distributed PCA is to find an eigendecomposition of **A** without having all the local datasets $A_{s}$ at a central site. The data are assumed to be centered, and if applicable scaled to unit variance which can be achieved easily using federated summary statistics. There are broadly two groups of algorithms, single-round approaches which communicate only once between the clients and the aggregator and iterative approaches which require multiple communication rounds. The single-round approaches follow the same main idea, but have different implementation details. Generally, a local summary statistic is computed and sent to the central aggregator, where it is merged to a global model.

### 2.5 Reconstitution of the covariance matrix

The reconstitution of the global (approximated) covariance matrix is a popular approach that has been implemented in different variations. They all rely on the observation that the covariance matrix can be computed exactly at the global server by adding up the local covariance matrices. This basic version is, for instance, used in Liu *et al.* (2018). In this version, the covariance matrices of the local datasets are computed and sent to the aggregator. At the aggregator, the local covariance matrices are summed up element wise. The eigendecomposition of this exact covariance matrix is computed and shared with the clients. We denote this version P-COV. A means to significantly reduce the transmission costs is to approximate the local subspaces and send these to the aggregator. In this case, a local singular value decomposition (SVD) is computed and the top-k eigenspace is sent to the aggregator, where *k* is fixed but arbitrary (Al-Rubaie *et al.*, 2017; Fan *et al.*, 2019; Imtiaz and Sarwate, 2018; Liu *et al.*, 2018; Qu *et al.*, 2002; Wang and Morris Chang, 2019; Won *et al.*, 2016). More precisely, in these algorithms, the local subspace $\left(V_{s}^{k},\Sigma_{s}^{k}\right)$ is computed at each site and sent to the aggregator (Algorithm 1, Lines 2 and 3). At the aggregator, a proxy covariance matrix $M_{s}^{p}=V_{s}^{k}\Sigma_{s}^{k}V^{⊤}$ for each site is reconstituted using $\left(V_{s}^{k},\Sigma_{s}^{k}\right)$, and added up element wise such that an approximation of the global covariance matrix $M^{p}$ is obtained (Algorithm 1, Line 8). As only a limited number *k* of eigenvectors is transmitted $M^{p}$ is an approximation of the hypothetical global covariance matrix. The global PCA is then computed by the eigendecomposition of the proxy covariance matrix (Algorithm 1, Line 12). This version is denoted AP-COV.

#### 2.5.1 Subspace aggregation

The federated PCA algorithm proposed by Balcan *et al.* (2014) computes a local subspace such as in the proxy covariance methods above but differs in the aggregation step. The local subspaces $\left(V^{k},\Sigma^{k}\right)$ are concatenated on the vertical axis and the singular value decomposition of this stacked subspace is computed (Algorithm 1, Line 10). It is conceptually the same as computing the proxy covariance matrix but more efficient depending on the dimensions of the input matrices. This version is called AP-STACK in the remainder of the article. Please refer to Algorithm 1 for a pseudocode description of these algorithms. (They have been merged due to their conceptual overlap.)

#### 2.5.2 Intermediate dimensionality

AP-COV and AP-STACK have a parameter k′ that determines the number of eigenvectors transferred to the aggregator. This number of intermediate dimensions k′ is usually larger than the target dimensions *k*, the size of the final subspace. Naturally, k′ determines the transmission cost of these approaches. This already hints at an issue regarding the dimensionality of the local subspaces: The number of retrieved eigenvectors is limited by the minimum dimension of the data matrix (i.e. either by the number of features, or by the number of samples) at the site. For instance, the subspace of a 20 × 20 000 matrix can only have 20 eigenvectors which means higher-order global subspaces, that is subspaces where the global *k* is set to be larger than any of the local *k* can possibly be, can suffer accuracy loss w. r. t. to the centralized solution.

Algorithm 1 Federated PCA using subspace aggregation

**Require:** Data matrices $A_{s}\in R^{n_{s}\times m}$, # eigenvectors *k*.

1: **Client**

2:   $U_{s},\Sigma_{s},V_{s}^{⊤}\leftarrow \mathrm{svd}(A_{s})$

3:   $\mathrm{send-to-aggregator}(V_{s}^{k⊤},\Sigma_{s}^{k})$

4: **Client**

5: **Aggregator**

6:   $\left[V_{s}^{k⊤},\Sigma_{s}^{k}]\leftarrow for\;s\in [S]\;\mathrm{get-from-client}(V_{s}^{k⊤},\Sigma_{s}^{k}\right)$

7:   **if** (P-COV, AP-COV) **then** ▹ Proxy cov. methods

8:

$$M\leftarrow \sum_{s}^{S}V_{s}^{k}\Sigma_{s}^{k}V_{s}^{k⊤}$$

9:   **else**▹ Balcan *et al.* (AP-STACK)

10:    $M\leftarrow \mathrm{stack-vertically}([\Sigma_{s}^{k}V_{s}^{k⊤}])$

11:   **end if**

12:   $U,\Sigma ,V^{⊤}=\mathrm{svd}(M)$

13:   $\mathrm{send-to-client}(V^{k⊤},\Sigma^{k})$

14: **Aggregator**

15:**Return**$V^{k⊤},\Sigma^{k}$ ▹ Return approximate subspace of $A^{⊤}A$.

### 2.6 QR-based PCA

Bai *et al.* (2005) propose a conceptually different method, which still only requires one communication round per participant. Their method is not designed for a star-like architecture, but could be considered for the cross-silo P2P architectures cited earlier (Beyan *et al.*, 2020; Warnat-Herresthal *et al*., 2021); therefore, we include it in the accuracy analysis. It can also be adapted to the star-like architecture by modifying the merge procedure. See Algorithm 2 for a pseudocode description of this algorithm. At the local sites *s*, a QR factorization of the data matrix is computed and $R_{s}$ is sent to the aggregator (Algorithm 2, Line 2). From the local QR factorizations, the global PCA is computed by stacking all $R_{s}$ matrices vertically to form $R\prime \in R^{n\times m}$. R′ is decomposed into Q,R″ (Algorithm 2, Lines 7 and 8). In the final step, the singular value decomposition of $R\prime \prime =U\Sigma V^{⊤}$ is computed and the top *k* eigenvector matrix $V^{k,⊤}$ is returned as the eigenvector of $A^{⊤}A$ (Algorithm 2, Line 9).

Algorithm 2 Federated PCA using QR factorization (Bai *et al.*, 2005)

**Require:** Data matrices $A_{s}\in R^{n_{s}\times m}$, # eigenvectors *k*.

1: **Client**

2:

$$Q_{s},R_{s}\leftarrow \mathrm{orthonormalize}(A_{s})$$

3:

$$\mathrm{send-to-aggregator}(R_{s})$$

4: **Client**

5: **Aggregator**

6:   $\left[R_{s}]\leftarrow for\;s\in [S]\;\mathrm{get-from-client}(R_{s}\right)$

7:   $R\prime \leftarrow \mathrm{stack-vertically}([R_{s}])$

8:   Q,R″←orthonormalize(R′)

9:   $U,\Sigma ,V^{⊤}=\mathrm{svd}(R\prime \prime )$

10:  $\mathrm{send-to-client}(V^{k⊤},\Sigma^{k})$

11: **Aggregator**

12: **Return**$V^{k}$ ▹ Return eigenvector matrix of $A^{⊤}A$.

### 2.7 Federated subspace iteration

Federated subspace iteration is a direct extension of the centralized subspace iteration (Halko *et al.*, 2011) and has been formulated in different versions (Balcan *et al.*, 2016; Hardt and Price, 2014; Pathak and Raj, 2011). It is the extension of power iteration which computes one vector at the time. Subspace iteration is described in Algorithm 3. Initially, a random eigenvector estimate $V_{i=0}$ is generated at the aggregator as the current eigenvector estimate and orthonormalized (Algorithm 3, Lines 1–3). The procedure then iteratively refines this estimate. It consists of a local phase and a global phase. In the local phase, the current candidate eigenvector matrix $V_{i-1}$ is multiplied by the covariance matrix of the local data to form $V_{s,i}=A_{s}^{⊤}A_{s}V_{i-1}$ (Algorithm 3, Lines 7–9). This candidate matrix $V_{s,i}$ is sent to the aggregator where the global estimate is computed by adding up the local eigenvector estimates $V_{s,i}$ element wise over the local estimates. The candidate eigenvector matrix is normalized using QR factorization and sent back to the clients (Algorithm 3, Lines 12–15). This procedure is repeated until convergence.

Algorithm 3 Federated Subspace Iteration

**Require:** Data matrices $A_{s}\in R^{n_{s}\times m}$, # eigenvectors *k*.

1: Generate $V_{0}\in R^{m\times k}$ randomly ▹ Initialize candidate eigenvector matrix of $A^{⊤}A$.

2: $V_{0}\leftarrow \mathrm{orthonormalize}(V_{0})$

3: i←1 ▹ Initialize iteration counter.

4: **while** termination criterion not met **do**

5:   **Client**

6:    $V_{i-1}\leftarrow \mathrm{get-from-aggregator}()$

7:    $V\prime_{s,i}=A_{s}V_{i-1}$ ▹ Update local eigenvectors $V_{s,i}$

8:    $V_{s,i}=A_{s}^{⊤}V\prime_{s,i}$

9:    $\mathrm{send-to-aggregator}(V_{s,i})$

10:   **Client**

11:   **Aggregator**

12:    [Vs,i]←get-from-client()

13:    $V_{i}=\sum_{s}V_{s,i}$ ▹ Add up $V_{s,i}$ element wise.

14:    $V_{i}=\mathrm{orthonormalize}(V_{i})$

15:    $\mathrm{send-to-client}(V_{i})$

16:    i←i+1

17:   **Aggregator**

18: **end while**

19: $V^{k}\leftarrow V_{i}^{k}$

20: **Return**$V^{k}$ ▹ Return converged eigenvectors of $A^{⊤}A$.

### 2.8 Vertical partitioning

In this article, we discuss the algorithms and applications of federated PCA for horizontally partitioned data. Please note that some applications in computational biology (for instance population stratification) require the decomposition of the sample-by-sample covariance matrix, which cannot be solved directly with every one of the algorithms evaluated in this manuscript. In the vertical case, the computation of the entire covariance matrix is not possible because for two sites *i* and *j* with *n_i_* and *n_j_* samples, respectively, only the partial covariance matrices $M_{i,i}\in R^{n_{i}\times n_{i}}$ and $M_{j,j}\in R^{n_{j}\times n_{j}}$ can be computed while the computation of $M_{i,j}\in R^{n_{i}\times n_{j}}$ and $M_{j,i}\in R^{n_{j}\times n_{i}}$ would require the transfer of the samples of site *i* to site *j*. To illustrate this, consider a gene panel as example. Every hospital measures *d* genes for their *n_i_* patients. At every site *s*, we can compute the gene-by-gene covariance matrix with the full dimensionality *d *×* d*, but we can only compute the partial patient-by-patient covariance matrices of $n_{s}\times n_{s}$ at each site without exchanging patient-level information. Nasirigerdeh *et al.* (2020) have shown that for federate GWAS pipelines, exchanging the entire sample eigenvectors potentially leads to a privacy breach where binary covariates of participants can be disclosed. Therefore, care has to be taken when exchanging the sample eigenvectors. In Hartebrodt *et al.* (2021), this problem is presented in greater detail, and an algorithm is developed, which solves this problem efficiently and without materializing the covariance matrix or exchanging the sample eigenvectors at all.

### 2.9 Other related methods

A plethora of algorithms has been designed for distributed sensor networks dealing with both horizontal data partitioning and vertical partitioning, including but not limited to work described in Bertrand and Moonen (2014), Fellus *et al.* (2015), Jelasity *et al.* (2007), Schizas and Aduroja (2015) and Wu *et al.* (2018). These algorithms cover cross-device FL. In contrast to cross-silo FL where large chunks of data are available at the sites, cross-device FL assumes a high number of devices such as sensors or mobile phones with limited compute power and relatively little data belonging to only one user. Due to the differing assumptions on architecture and computational resources, and the frequent use of (randomized) P2P communication, algorithms for this use case will not be considered here. Chen *et al.* (2021) describe a gradient method that uses matrix deflation for the computation of more than one eigenvector which is impractical due to the increased communication effort (cf. Hartebrodt *et al.*, 2021).

### 2.10 Test setup and metrics

In the optimal case, the federated PCA produces exactly the centralized solution. In order to estimate the performance of the algorithms on the realistic data from TCGA, we simulate the execution of the federated algorithm with the data distributed according to the TSS as described above. Since some of the sample sites are quite small, in a second experiment, we additionally group several sample sites together to form larger ‘meta-sample-sites’ of approximately the same size each using a greedy heuristic. We chose this strategy to better investigate approaches that compute local subspaces. As explained above, the dimension of such a subspace is strictly limited by the number of samples.

To evaluate the algorithms’ performance, we compare the result of the federated PCA to the solution computed on the centralized data. As a reference implementation, we use the implementation in scipy.sparse.linalg which internally uses the LAPACK package. The comparison of the models is done by calculating the angles between the leading respective eigenvectors of the centralized and the federated solution. The angle is calculated as $\theta =\text{co}{\text{s}}^{-1}\frac{x\cdot y}{\left||x||\cdot ||y|\right|}$ with **x** and **y** the reference and federated vector, respectively. The angle is transformed from radians to degree. We chose the angle between the eigenvectors over the data reconstruction error, because the ‘loadings’, the coordinates of the eigenvectors, are routinely used in gene expression analysis, for example to detect correlated genes (Fehrmann *et al.*, 2015). Therefore, the individual coordinates of the eigenvectors must be taken into account when comparing the resulting subspaces. For applications which only rely on the projected data, and not on the individual loadings, we compute the data reconstruction error. The data reconstruction error is the distance of the original data from the reconstructed ‘denoised’ data. It is defined as $|AV^{k}V^{k⊤}-A{|}_{F}$. It is obtained by computing the projections of the data onto the first *k* eigenvectors to obtain the principal components (PCs) and then reprojecting the PCs using the transpose of the eigenvectors to reconstruct data of the original dimensionality.

Complementing the simulated results which established the accuracy of the methods, we also provide a real implementation of the algorithms. We use the FeatureCloud (Matschinske *et al.*, 2021) platform for this purpose and implemented an app that can compute all previously presented algorithms. The application has multiple modes, including a batch mode and a train/test mode allowing for cross-validation splits. We then set up a test using the FeatureCloud ‘Testbed’, which allows to simulate a federated setting by spawning multiple clients on the same machine. The parameters are passed via a remote relay server, meaning the transmission cost is close to a realistic estimate. For more details on this system, please refer to the website featurecloud.ai and the publication (Matschinske *et al.*, 2021). We measure the wall clock time, the number of iterations and the number and size of sent packages. The tests were run on a UNIX server with 502GB RAM and 64 CPUs partially in parallel. AP-COV has been omitted because it is as accurate as AP-STACK and has the same communication properties as P-COV. We used a randomly generated dataset with 5000 samples and 10 features, and the MNIST dataset. They were randomly chunked into three, and five equal chunks and repeated five times. We set the termination criterion to $1e^{-9}$ for SUB-IT. Table 2 summarizes the investigated parameters.

**Table 2.** The parameter choices for the truly federated implementation of PCA

Parameter	Choices
Algorithm	P-COV, AP-STACK, SUB-IT, QR-PCA
Clients	3, 5
Datasets	Random, MNIST
Epsilon	$10e^{-9}$

### 2.11 Practical application using integrated Psoriasis data

A possible use of PCA is the visualization of the data to detect batch effects, systematic shifts in the data distribution due to different experimental processing of the data. We illustrate this use case with a publicly available collection of Psoriasis datasets (Federico *et al.*, 2020). The studies were originally not conceived as a federated study, but have been manually curated and preprocessed following the same computational pipeline (Federico *et al.*, 2020). We selected the sequencing datasets with accession numbers GSE107871, GSE123785, GSE41745, GSE54456, GSE67785, GSE83645, GSE117405, GSE123786, GSE47944, GSE63979 and GSE74697. We investigate the differences of federated exact PCA (SUB-IT), federated approximate PCA (AP-STACK) and the naïve superimposition of the local PCA spaces to show whether they can be used to accurately determine the presence of batch effects in the data. We furthermore show how a sampling strategy can be used to avoid sharing the exact projections and still be used to detect the batch effects. The procedure works as follows: first, the eigenvectors are computed using exact federated PCA (e.g. SUB-IT). Then, the data are projected onto the PCs. Instead of sharing the projections, we compute the empirical covariance matrix of the projections locally, and sample artificial data points from a multivariate Gaussian distribution. These artificial projections are sent to the other participants, which obtain an idea of the data layout. We include a pseudocode description of this procedure in the Supplementary Algorithm S1.

## 3 Results

### 3.1 Analysis of the exchanged and final parameters

Firstly, we analyze the actually transmitted information and investigate which algorithm discloses the highest amount of information. *V* denotes the complete eigenvector matrix and *V^k^* and $V^{k\prime }$ the matrices containing the top *k* and k′ eigenvectors, respectively. Table 3 summarizes the parameters known to the clients and the aggregator at the end of the run. Note that *V* allows the computation of the covariance matrix *M* and *V^k^* and $V^{k\prime }$ analogously its approximations *M^k^* and $M^{k\prime }$. Given the aggregator has all the exact local covariance matrices it can run local subspace iteration and therefore access $V_{i}^{k}$ when using P-COV and QR-PCA, given the same initialization. Following this reasoning, we claim that in terms of disclosed knowledge, P-COV and QR-PCA are equivalent and disclose the highest amount of information. AP-COV and AP-STACK disclose larger *V^k^* subspaces than SUB-IT which only discloses the required *V^k^*; however, SUB-IT might be prone to iterative leakage and disclose the covariance matrix. It is outside the scope of this manuscript to try and attack either algorithm. It is also apparent that there is an asymmetry of the knowledge of the parameters in the chosen architecture which favors the aggregator who gains knowledge of all intermediate steps. This asymmetry is due to the chosen architecture and can be trivially resolved by adopting a P2P architecture at the expense of increased network traffic. Another solution to this problem is the use of secure multiparty aggregation (Cramer *et al.*, 2015) which can be used to hide parameters with an additive aggregation strategy. This applies to P-COV, AP-COV and SUB-IT but not trivially to QR-PCA and AP-STACK which use QR decomposition and singular value decomposition as their respective aggregation strategies.

**Table 3.** Summary of the transmitted parameters and the computable parameters

Algorithm	*V^k^*		$V^{k\prime }$		*V*		*R_s_*		$V_{i}^{k}$	
	C	A	C	A	C	A	C	A	C	A
P-COV	s*	S	s*	S	s*	S	s*	S	s*	S
AP-COV	s*	S	s*	S						
AP-STACK	s	S	s	S						
SUB-IT	s*	S							s*	S
QR-PCA	s	S	s	S	s	S	s	S	s	S

*Notes*: *s* denotes the knowledge of the local parameter, *S* denotes the knowledge of all local parameters and hence the aggregate. A * indicates which parameters can be hidden via the use of secure addition.

### 3.2 Network traffic

Here, we analyze the communication requirements for federated PCA. Let D be the dimensionality of the parameters in terms of floats transmitted between client and aggregator and let N be the number of communication rounds. Let T be the total transmission cost. Recall that the global data matrix has dimensions $A\in R^{n\times d}$ and is divided into *S* local data matrices *A_s_* with *n_s_* samples and *d* dimensions each. *k* is the number of eigenvectors of the final decomposition and k′ the intermediate dimensionality if applicable. *i* is the number of iterations for subspace iteration to converge. Table 4 summarizes the parameter and the associated transmission cost exchanged between the sites. All methods assume a centered data matrix, so the exchange of the column sums and the number of samples is required.

**Table 4.** Summary of the transmitted parameters and the required number of iterations

Algorithm	Param.	Direction	D	N	T
P-COV	**M**	C → A	*d *×* d*	1	$O(d^{2})$
	$V^{k}$	C ← A	*d *×* k*	1	
AP-COV	$V^{k\prime }$	C → A	*d *×* k*	1	O(dk′)
	$V^{k}$	C ← A	*d *×* k*	1	
AP-STACK	$V^{k\prime }$	C → A	*d *×* k*	1	O(dk′)
	$V^{k}$	C ← A	*d *×* k*	1	
SUB-IT	$V^{k}$	C ↔ A	*d *×* k*	i	O(dki)
QR-PCA	**R**	C → A	*d *×* d*	1	$O(d^{2})$
	$V^{k}$	C ← A	*d *×* k*	1	

*Note*: C, client; A, aggregator.

### 3.3 Accuracy on a standard image dataset

To put the performance of the algorithms into perspective, we first provide accuracy values for the performance on the standard image dataset MNIST. This dataset consists of 60 000 gray scale images containing 784 pixels each. The dimensionality is hence *d *<* n*. Here, the performance of the algorithms is generally very good. Table 5 summarizes selected angles for each of the selected approaches using the MNIST dataset split into 20 equal chunks. Using these data, all algorithms lead to a good approximation of the subspace with low angular deviations. SUB-IT, P-COV and QR-PCA outperform AP-COV and AP-STACK, but by a small margin.

**Table 5:** Comparison of the PCA performance using the MNIST dataset over 20 randomized splits, the TCGA data distributed according to the tissue sample site and combined into 2 and 5 meta-sites, respectively

EV	PCA	MNIST	TCGA	5	2
1	Exact	0	0	0	0
	Approx	0.47	13.0	10.4	6.79
5	Exact	0	0	0	0
	Approx	1.46	38.9	45.0	32.9
10	Exact	0	0	0	0
	Approx	10.35	71.3	69.3	46.7

*Notes*: Exact methods are SUB-IT, QR-PCA and P-COV, approximate methods are AP-COV and AP-STACK. Shown are the angles between the federated eigenvector and references for 1st, 5th and 10th eigenvectors, respectively.

### 3.4 Accuracy of the selected approaches to TCGA data

Table 5 summarizes the accuracy of the eigenvectors computed using the simulated federated PCA using TCGA data. As a measure of quality, we show the angle between the eigenvector calculated by the federated approach against the centralized singular value decomposition. There are three values, one for the original data partitioning extracted from TCGA, and one each for the ‘meta-sample-sites’. Generally speaking, SUB-IT, as well as the P-COV and QR-PCA perform accurately regardless of the data distribution. The angle between all selected eigenvectors is close to 0. The approximate algorithms AP-COV and AP-STACK do not perform as well. They improve when creating larger meta-sites which confirms that these algorithms only perform well, when there are sufficiently many samples at each collection site. In order to put these values into perspective, in Table 6, we also provide the ratio of the subspace reconstruction error achieve by the federated method divided by the gold standard reconstruction error. The deviation of the data reconstruction error of the federated solution w. r. t. centralized solution is small. This indicates that downstream analyses relying on the coordinates of the eigenvectors are likely to suffer from the approximate approaches, while analyses merely relying on proxy data are more resilient against the errors introduced by the approximations. Supplementary Figure S4 shows an extended view of these results.

**Table 6.** Comparison of the PCA performance

EV	PCA	MNIST	TCGA	5	2
1	Approx	1	1	1	1
5	Approx	1.000022	1.006	1.005	1.002
10	Approx	1.000101	1.01	1.008	1.002

*Notes*: Shown are the ratios data of the reconstruction errors of data projected using the federated eigenvector and references for 1st, 5th and 10th eigenvectors, respectively, for AP-COV/AP-STACK only.

### 3.5 Application of federated PCA to Psoriasis data

**Fig. 2.**
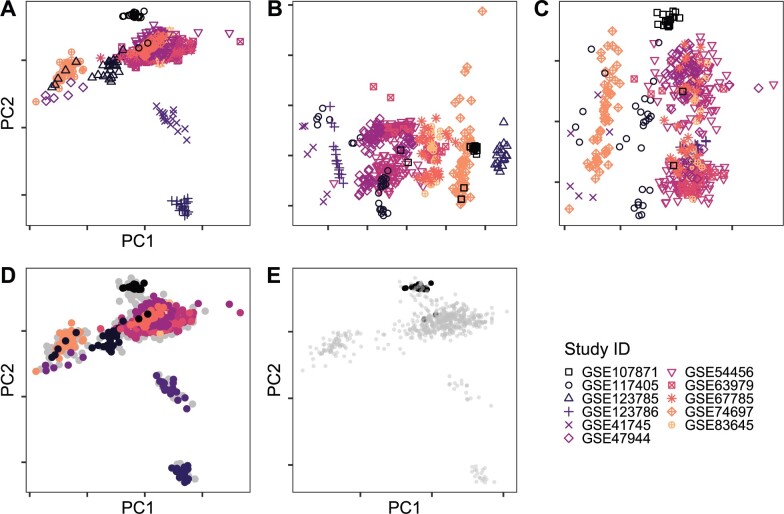
Comparison of centralized PCA/SUB-IT (**A**), combined local projections (**B**) and AP-STACK (**C**) on Psoriasis data (GEO accession numbers in the figure). The pattern for the exact PCA is markedly different from both the naïve combinations PCA and the approximate PCA. Contrary to approach (A), (B) and (C) are not suited to detect the batch effects present in the data. (**D**) The exact and resampled projections jointly. (**E**) A local view of one study. The client can get an understanding of how their samples fit into the global context

We applied the methods on a manually curated multicentric Psoriasis dataset where individually performed studies were assembled and reprocessed with the same computational pipeline. In Figure 2, we show the PC plots of the data using federated power iteration (SUB-IT), which is identical to the centralized power iteration, AP-STACK and by superimposing the results of the local computations. The exact PCA (SUB-IT) shows that there are prominent batch effects in the data, as the data separates according to the experiment, whereas this is not replicated by AP-STACK and the superimposition of the locally computed PCAs. This is visualized in Figure 2A–C. The additional plots visualize the sampling-based visualization approach. Figure 2D shows all exact and sampled projections. Generally, the sampled projections and the exact projections overlap. Figure 2E shows the view, one of the participants would have. In gray, the resampled projections show the global sample distribution, whereas the black dots represent the local, exact projections, which were never shared. In Supplementary Figure S2, we show similar results for simulated data. Supplementary Figure S5 shows more individual PC plots in comparison with the global result. We also include plots that visualize the distributions of covariates in the data (Supplementary Fig. S6). Further figures show similar results for federated PCA on single-cell data using different *k* for the visualization (Supplementary Figs. S7 and S9) and downstream analyses such as clustering (Supplementary Fig. S10 and Table S2) and gene importance scoring (Supplementary Fig. S11).

### 3.6 Practical implementation

Table 7 shows the results of the empirical runtimes of the PCA algorithms for different datasets averaged over five runs. AP-STACK, P-COV and QR-PCA have low execution times in the order of seconds. The low number of executions does not allow to rank the algorithms further. SUB-IT has longer execution times and requires more data transmission. Additional results for random data can be found in Supplementary Table S1.

**Table 7.** Results of the federated test runs using MNIST data

Dataset	Algorithm	Sites	Time[s]	Iter.	MB
MNIST	P-COV	5	27	1	10 555
		3	20	1	21 118
	AP-STACK	5	20	1	649
		3	25	1	325
	QR-PCA	5	23	1	11 242
		3	30	1	5626
	SUB-IT	5	1208	1000	296 073
		3	1090	1000	148 180

## 4 Discussion

### 4.1 Choice of the performance criteria

The angle between the eigenvectors is a very stringent criterion for the performance of the algorithm, as with high-dimensional vectors very few deviating coordinates can lead to a drastic change of the angle. The data reconstruction error measures the distance between the reconstruction of the data and the actual data and is therefore suitable for analyses that solely rely on proxy data. The ratios of the subspace reconstruction errors show that both bases (federated and centralized) lead to a reasonable numerical solution but can be misleading for the downstream data analysis; therefore, we report the angle between the eigenvectors. The wall clock time and number of communication steps are both useful measures as their combination allows to estimate the run times for future studies.

### 4.2 Analytical performance of the methods

Approximate methods generally perform poorly according to the angular deviation of the eigenvectors, especially when retrieving many eigenvectors. This strong deviation of the federated eigenvectors from the centralized baseline implies that the analysis of the loadings of the eigenvectors is not possible using the approximate methods and is additionally very vulnerable to outliers. The eigenvector coordinates may deviate significantly even in low ranks, therefore different hypotheses will be generated using approximate PCA compared to centralized PCA. This is further illustrated in Figure 2 where the results of exact and approximate PCA are fundamentally different, and the approximate method does not reproduce the result to an extent, where the batch effect in the data cannot be detected. According to the subspace reconstruction error for the TCGA data, most methods perform reasonably well even on unfavorably distributed data with many small subsets. However, due to the centralized processing, TCGA data are still likely to give optimistic estimates of the error incurred due to federated approximate PCA, meaning that the sites do not show a fundamentally different data distribution.

In the Supplementary Material, we include additional analyses which show that the approximate method induced differences in the downstream analyses of single-cell data, such as cluster assignment. Furthermore, the method is vulnerable to outliers, which is not surprising given the general vulnerability of PCA to outliers. This also impacts popular visualization methods such as UMAP. It has recently been argued that UMAP and t-SNE should only be used for the coarse analysis of the data (Chari *et al.*, 2021). If that is the case, the approximate methods could be used for visualization of datasets larger than the ones studied here.

### 4.3 Computational performance of the methods

Our practical implementations show that the overhead through the use of the federated methods is acceptable given the long sourcing process of biological data and the potential privacy gain. The FeatureCloud platform introduces a certain overhead through virtualization and encryption techniques. Nonetheless, with the run time of a few seconds to a few minutes for federated PCA researchers can realistically use these methods in practice. The bottleneck is the number of communication steps required; therefore, federated power iteration is slower than the single-round methods. The advantage of SUB-IT is that it can cope with a higher number of features while performing exact PCA, contrary to QR-PCA and P-COV which need to load the covariance matrix into memory. The convergence criterion for power iteration is also set very stringent, so a lower number of iterations and thus a decreased run time might be sufficient in practice.

### 4.4 Privacy of federated SVD

Critiques may raise the issue of the privacy of the parameters. Indeed, the amount of data transmitted between the sites is quite large since they are the result of a matrix decomposition. Recall that we are working on the matrix $A\in R^{n\times d}$, where *n* is the number of samples and *d* is the number of features and $A=U\Sigma V^{⊤}$. This means each vector *u* in $U=[u_{1},u_{2},\ldots ,u_{k}]$ contains elements belonging to the samples, whereas $V=[v_{1},v_{2},\ldots ,v_{k}]$ summarizes the features across all samples. If all participants are to receive the complete SVD, then the aggregator has to broadcast the final *U* and *V* to all the clients. In previous work, it has already been shown that federated pipelines which include the use of the sample eigenvectors *U* are prone to data leakage when broadcasting the full eigenvectors. Therefore, we highly recommend to not broadcast the sample-specific eigenvectors *U*. Ideally, this would happen in an oracle fashion, where the parties gain knowledge of the output, but none of the intermediate parameters. Unfortunately, this is not the case, therefore in Section 3, we established a hierarchy of the approaches in terms of trivially reconstructable data and parameters.

Several articles discuss privacy-preserving PCA or power iteration in a federated setting via encryption and secure multiparty computation techniques (Al-Rubaie *et al.*, 2017; Cho *et al.*, 2018; Pathak and Raj, 2011; Rathee *et al.*, 2018; Won *et al.*, 2016) or differential privacy (DP; Balcan *et al.*, 2016; Hardt and Price, 2014; Imtiaz and Sarwate, 2018; Wang and Morris Chang, 2019). A few articles (Liu *et al.*, 2020; Won *et al.*, 2016) assume that the aggregated covariance matrix is private. The authors of Pathak and Raj (2011) assume that the aggregated eigenvector updates are private. Generally speaking, if the aggregated parameters are considered private, since the methods generally only require additive aggregation, secret sharing by sharding the data or using fixed-point arithmetic, can be added with relatively little overhead (Cramer *et al.*, 2015). A protocol that does not use the clear-text covariance matrix has been proposed by Al-Rubaie *et al.* (2017) who use a garbled circuit protocol that allows to compute the eigenvectors securely based on the homomorphically aggregated covariance matrix. The latter approach is quite time intensive on small datasets in simulation (Rathee *et al.*, 2018) introduces improved primitives required for PCA using homomorphically encrypted centralized data. The empirical evaluation unfortunately does not include data at the scale of high-dimensional biological data (*d *=* *20 is the largest dimensions) and a realistic number of iterations (*i *=* *5 is the total number of iterations). The extension to the federated setting provides an additional challenge. The protocol in Cho *et al.* (2018) requires the participants to shard their data and send them to two external parties. The communication overhead is quite large. The high dimensionality of the data is an obstacle for DP as the noise scales with the number of variables. Since the number of variables is large, these approaches cannot be used without severe degradation of the results. Furthermore, the choice of a good *ϵ* is not easy in practice. A major obstacle in the adoption of these techniques is the lack of ready-to-use libraries implementing the methods. We expect the technical challenges to be resolved in the near future, through more efficient protocols and ready-to-use implementations. However, even if it is possible to retrieve the eigenvectors privately, most of the downstream analyses in bioinformatics use the projections of the data onto the eigenvectors. Therefore, even differentially private eigenvectors are not sufficient to ensure privacy. (Due to the use of the data for the projection, this operation does not fall under the closure under post-processing.)

### 4.5 Choice of an appropriate algorithm

**Fig. 3.**
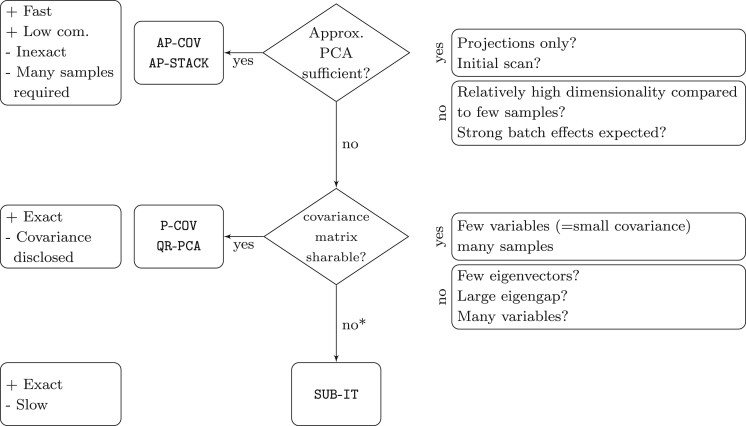
Decision help for researchers intending to use federated PCA. *It is likely that with sufficient iterations the covariance matrix may be reconstructed

In the following section, we will give guidelines for the choice of an appropriate algorithm which we summarize in Figure 3. Due to the various considerations in a federated study, not every algorithm is appropriate for every setting. Nonstar-like architectures can achieve $O(log_{2}(n))$ communication steps when using single-round approaches with *n* the number of clients. In terms of data disclosure and storage requirements, which amounts to the entire covariance matrix, the methods are equal. Since the aggregation method in QR-PCA is a QR factorization for which secure aggregation is not immediately possible, P-COV should be preferred when secure aggregation is required. If an approximate eigenvector is sufficient, AP-COV and AP-STACK are useful. However, only AP-COV allows secure aggregation because AP-STACK uses SVD as its aggregation method. Furthermore, AP-COV and AP-STACK depend on a high number of samples to achieve good results and may fail catastrophically in practice when data are limited or confounded. SUB-IT is exact and only discloses *V^k^* to the aggregator given a limited number of iterations. Our practical implementations show that all methods can achieve reasonable run times. The potential data leakage induced by subspace iteration remains an open question and will be subject to further research.

### 4.6 Other recommendations

In addition to choosing an appropriate algorithm, we suggest to carefully consider the information that is required for downstream analyses. Notably, restricting the number of eigenvectors *k* keeps the amount of communicated data and the number of communication steps low. It is possible to retrieve further eigenvectors should this be required later on. Before the use of the federated tools, it should be considered whether all local datasets need to be cleaned from obvious outliers and it should be assessed whether the populations at the clients are eligible for joint analysis. The inspection for outliers and their removal are necessary, because outliers have a disproportional effect on the PCA. In practice, the presence of outliers in the PCA can warrant a recomputation of the PCA and lead to unnecessary information disclosure if the outliers could have been detected beforehand. On the other hand, local outliers may not be outliers globally. In this case, the sampling approach can be chosen, where instead of using the original data, artificial data points are generated. The artificial data points act as representatives and allow the clients to assess global patterns in the data without seeing data from other clients. For instance, a client may have a very small number of points belonging to a cluster, so they appear as outliers locally. In the global context, they lie within a larger cluster and should not be removed. Using the artificial points this can be verified without breaching confidentiality. Users should evaluate, whether it is deemed a privacy breach, if the other participants know that there are client-specific groups, which could be vulnerable. A local PCA can also be performed to assess the information content of the components, including the computation of the eigengap, an indicator for the convergence behavior of subspace iteration (the larger the eigengap, the quicker the convergence). Overall, the users need to assess what the desired outcome of the analysis is, e.g. the detection of batch effects or groups, or the creation of lower-dimensional data, and consider whether the summary statistics communicated in this context could create privacy breaches. The nature of biomedical analyses is to gain a general understanding of the data. PCA can be a way to gain this understanding, but it discloses a high amount of information in the process (the variable means, variances, the covariance matrix and/or the eigenvectors, and if applicable the projections). The answer to whether this is acceptable cannot be given in a general way.

## 5 Conclusion

In this article, we identified existing methods for federated PCA, evaluated them using a realistic non-iid setting as well as random data distributions and provide a practical illustration of its application using transcriptomics data. Importantly, we implemented the methods and benchmarked their run time, providing valuable information for the applicability of the algorithms in a real setting. Additionally, we provided easy to follow guidelines for the future users to select the most appropriate algorithm and highlight important considerations before conducting a federated analysis.

The nature of PCA requires a high amount of information to be exchanged in the process of computation. The intermediate parameters can be masked by the use of appropriate secure computation methods. However, the final result is likely to be broadcast to the participants of a federated study. Given that the goal of the analysis is to gain a general understanding of the data, a privacy breach would manifest, if it was possible to infer sufficient information on individuals in the datasets from the shared results. Therefore, in the future it needs to be investigated under which conditions the communication of certain information (such as the projections) should be prohibited; or if perturbation methods must be used at the cost of accuracy. Data analysts will need to weigh the privacy versus the utility of the analysis in a case-by-case manner.

## Supplementary Material

vbac026_Supplementary_Data

## Data Availability

The processed Psoriasis data is available at 10.5281/zenodo.4009497 (Federico et al., 2020). The studies used in this article have the accession numbers GSE107871, GSE123785, GSE41745, GSE54456, GSE67785, GSE83645, GSE117405, GSE123786, GSE47944, GSE63979 and GSE74697. The MNIST data set can be downloaded from http://yann.lecun.com/exdb/mnist/.
