# Supplementary material for: Federated horizontally partitioned principal component analysis for biomedical applications
Source: Bioinform Adv. 2022 Apr 26;2(1):vbac026. doi: 10.1093/bioadv/vbac026 (PMC9710634; doi:10.1093/bioadv/vbac026)
Supplement: vbac026_Supplementary_Data [file vbac026_supplementary_data.zip › supplement.pdf]

Supplementary Material to Federated Horizontally Partitioned Principal Component Analysis for Biomedical Applications

Anne Hartebrodt* and Richard Röttger*

April 14, 2022

1 Visualization of the sample distribution in TCGA according to TSS

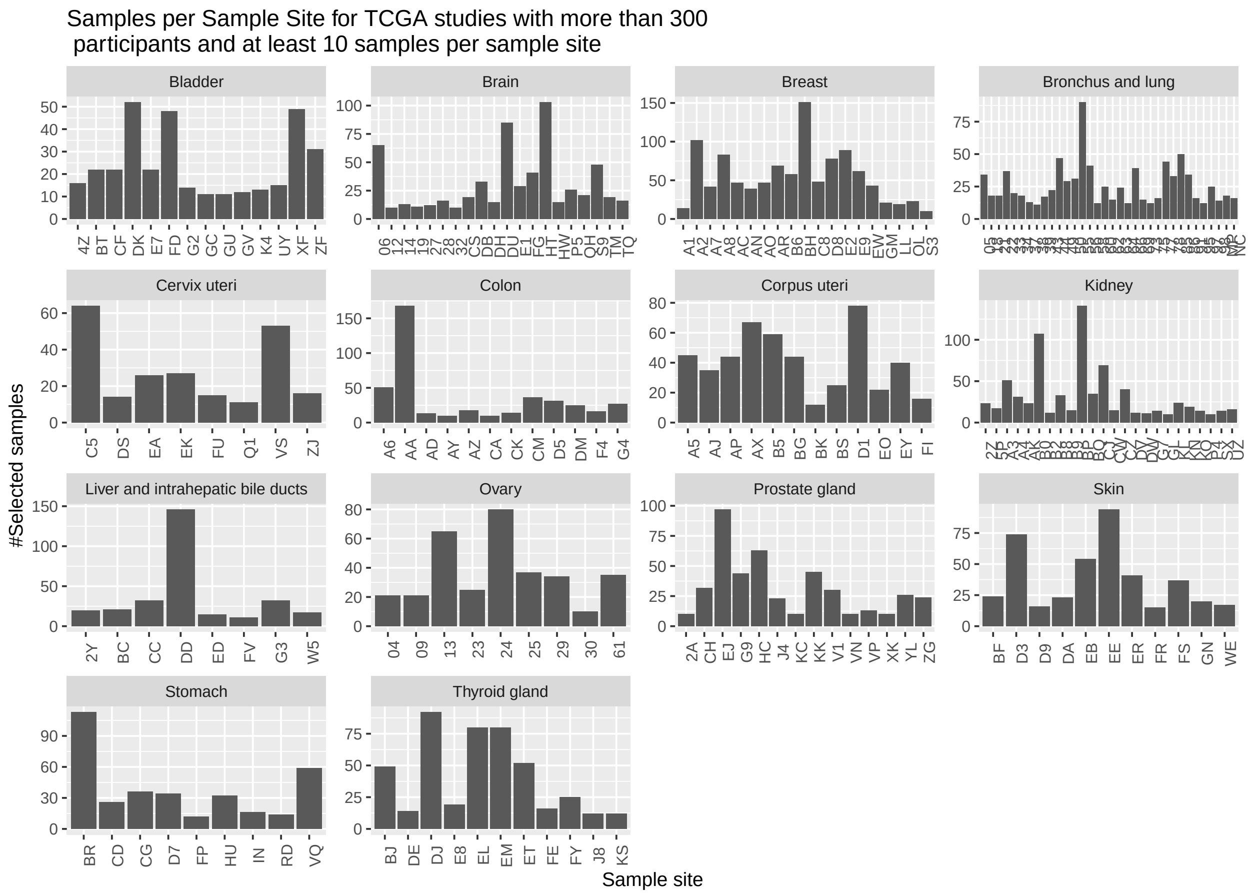

Figure 1: Distribution of samples over the tissue collection sites for different cancer types. The data was downloaded from TCGA. Selected studies had to contain at least 300 participants and study sites with fewer than 10 samples were excluded.

*Department of Mathematics and Computer Science, University of Southern Denmark, Campusvej 55, 5230 Odense, Denmark

1.1 Supplemental Algorithm – Data sampling

In order to prevent potential attacks on the data using the projections, we suggest to not send the original projections. Instead, projections can be sampled based on the eigenvectors and the local summary statistics. The global behaviour of the data is retained, but the indentification of individual data points can be prevented. The reidentification of data points is called a membership inference attack [4]. This can happen if attackers have access to parts of the measurements and want to check, if specific samples have been used in a trial. Since, one can sample infinitely many similar samples from the covariance matrix, this would make it impossible to say if a sample was part of the analysis or not based on the projections.

Here, we describe the generation process in more detail: Firstly, we compute the exact eigenvectors using for instance power iteration (line 1). We suggest to use the exact eigenvectors, to prevent misrepresenting the global axes of variation in the data, which we have shown is likely with approximate methods. Then, the local data is projected onto the first k principal components (line 3. Using the projected data, we compute the covariance and the mean of the projections lines 4 to 5. These represent 'localized' summary statistics which reside only at the clients. Using these summary statistics, we recreate artificial data points which are sent to the aggregator and redistributed to the clients (lines 9 to 12). The clients can then use the sampled projections, together with their exact projections to see, if their data follows the global trend, or if they have outliers. This is done to detect batch effects. If the knowledge of a batch (or subgroup) at a client is sensitive information, naturally this technique can not be used.

Algorithm 1 Federated Data Visualization

Require: Data matrices $\mathbf{A}_s \in \mathbb{R}^{n_s \times m}$, # eigenvectors k. ▷ Run federated PCA, e.g. federated subspace iteration

1: $\mathbf{V}, \Sigma \leftarrow$ `federated-subspace-iteration`()
2: **Client**
3: $\mathbf{P}^s \leftarrow \mathbf{A}^s\mathbf{V}$ ▷ Project the local data
4: $\mathbf{M}^s \leftarrow \mathbf{P}^{s\top}\mathbf{P}^s$ ▷ Compute the local covariance matrix of the projected data
5: $\mu^s \leftarrow Mean(\mathbf{P}^s)$ ▷ Compute the mean of all features of the local projections
6: $\mathbf{P}'s \leftarrow\sim \mathcal{N}(\mu^s, \mathbf{M}^s)$ ▷ Sample n_s artifical points to replace the local projections $\mathbf{P}$ using the empirical mean and the covariance
7: **Client**
8: **Aggregator**
9: $P \leftarrow$ `get-from-client` $\mathbf{P}^{'s}$ ▷ Aggregate all local subsampled projections
10: **Aggregator**
11: **Client**
12: $\mathbf{P}' \leftarrow$ `get-from-aggregator` $\mathbf{P}'$
13: Visualize $\mathbf{P}'$ and $\mathbf{P}^s$ jointly. ▷ Visualize the global, sampled projections, and the local exact projections together to locally identify outliers or batch effects.
14: **Client**

1.2 Application of federated PCA to simulated count data

In order to put the accuracy results into perspective, we use simulated count data for an empirical evaluation of these results and the influence of batch effects on the methods. We use the package splatter [10] to simulate group and batch effects simultaneously. In splatter, a batch effect is defined as a systematic error applied to all the genes in a batch, whereas a group effect pertains only to a subset of differentially expressed genes. We use data with 5 batches containing 500 cells each and 2 expression groups. We simulated 1 data set where the group effect is the stronger effect and 1 data set where the batch effect is the major driver of the variance in the data. We then computed exact and approximate federated PCA using the batch variable as a surrogate for the study site. In the supplement, we also illustrate the performance for the PCA methods using real data which has been artificially split into sites providing more downstream analyses.

Figure 2 shows a comparison of centralized, approximate (`AP-STACK`) and exact federated PCA (`SUB-IT`) using two different simulated data sets. The first data set contains simulated count data without strong batch

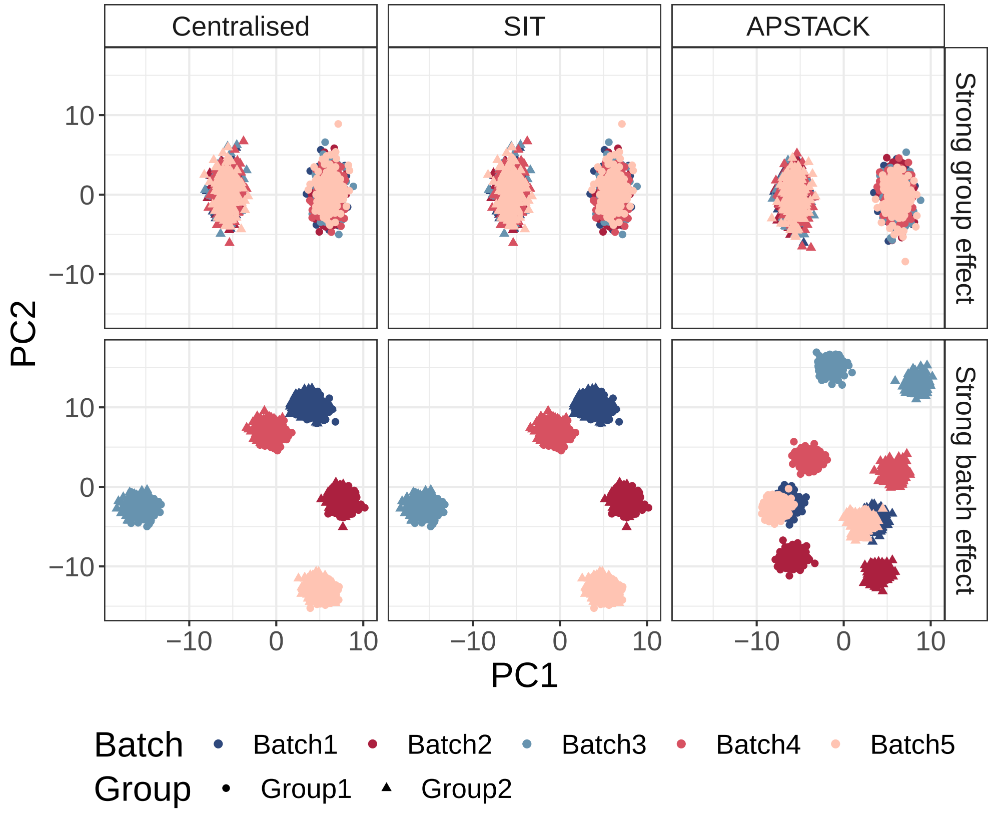

Figure 2: Comparison of centralized PCA, `SUB-IT` and `AP-STACK` using simulated single cell count data. The upper panel depicts data where the group (e.g. case-control) is the strongest variance driving effect across all sites. The lower panel shows data with a strong batch effect. `AP-STACK` is not able to reconstruct the embedding faithfully with strong batch effects.

effects, meaning that the group effect is the major driver of variation in the data. In this scenario, both federated methods reconstruct the low dimensional embedding faithfully to the centralized algorithm which clusters the data according to the group label. The second data set contains count data with a simulated batch effect that is stronger that the group effect. The centralized algorithm clusters the data according to the batch variable. `SUB-IT` computes the same representation, whereas `AP-STACK` separates most, but not all of the batches into separate groups and the spatial layout of the batches is different.

2 Drop out experiments

It is a known problem of centralized principal component analysis that it is vulnerable to outliers [8]. Outliers are data points which deviate strongly from the overall trend. Since PCA is a variance based linear decomposition of the covariance matrix, single data points can have a very strong influence on the eigenvectors. The fewer samples are available, the higher the impact of an outlier. In order to quantify this problematic with respect to our quality criterion the angle between the eigenvectors, we include an simulation, where we drop single data points from the data. Specifically, we performed the following computations: The exact PCA of a data set was computed. Then a random sample was removed from the data and the exact PCA was computed again. The angle between the original eigenvector and the 'dropout' eigenvector was computed. Figure 3 shows the effect of dropping single samples from the data and recomputing the PCA. From the box plots it can be seen that most samples do not lead to a strong deviation of the eigenvector from the original eigenvector, but some distort the result strongly. Therefore, in a low sample regime, the approximate methods do not perform well.

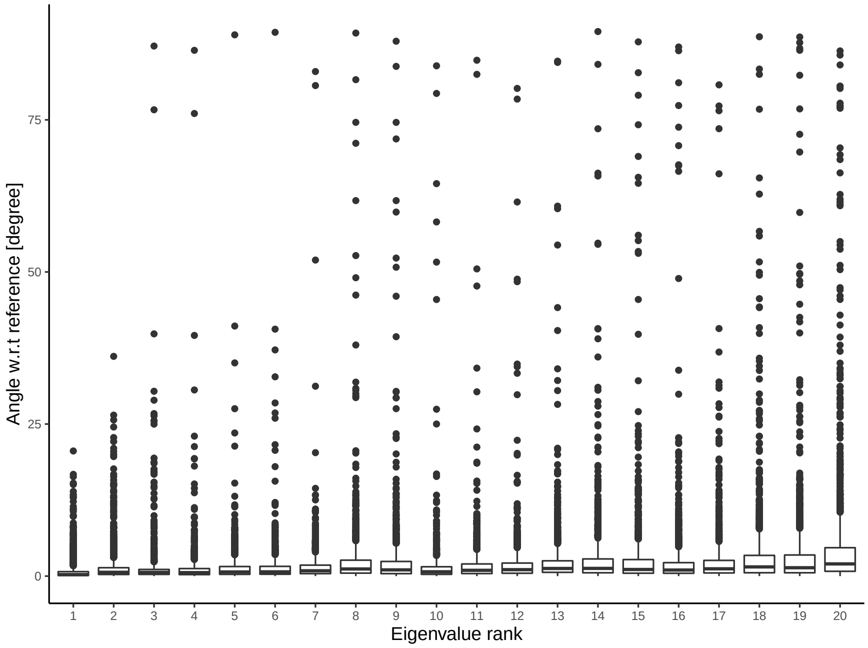

Figure 3: [Power iteration] Angles with respect to the gold standard when leaving out an individual sample. The plot presents aggregated statistics over all the data sets from TCGA, but all the cancer types were treated as individual data sets.

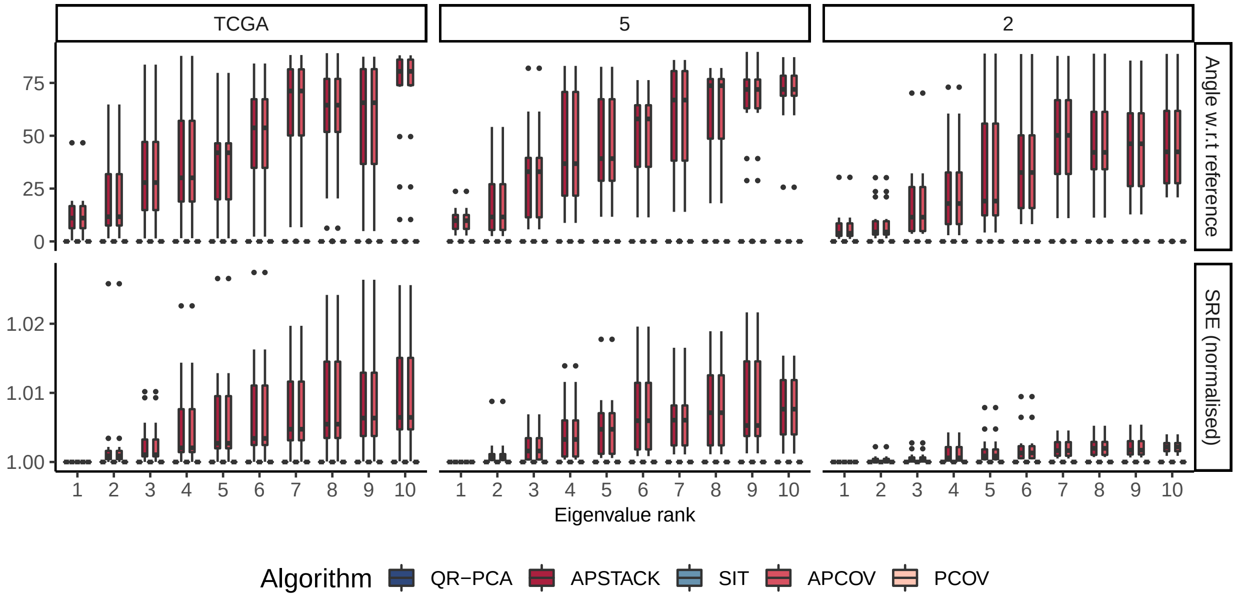

Figure 4: Extended PCA accuracy plot: Comparison of the different PCA algorithms w. r. t. to the angle between the leading eigenvectors with the TCGA data distributed according to the tissue sample site, and combined into 2 and 5 meta sites respectively. `SUB-IT`, `QR-PCA` and `P-COV` achieve perfect accuracy both according to the angle between the eigenvectors (upper panel) and the subspace reconstruction error (SRE). With higher rank, the accuracy of the proxy method deteriorates (`AP-STACK`, `AP-COV`).

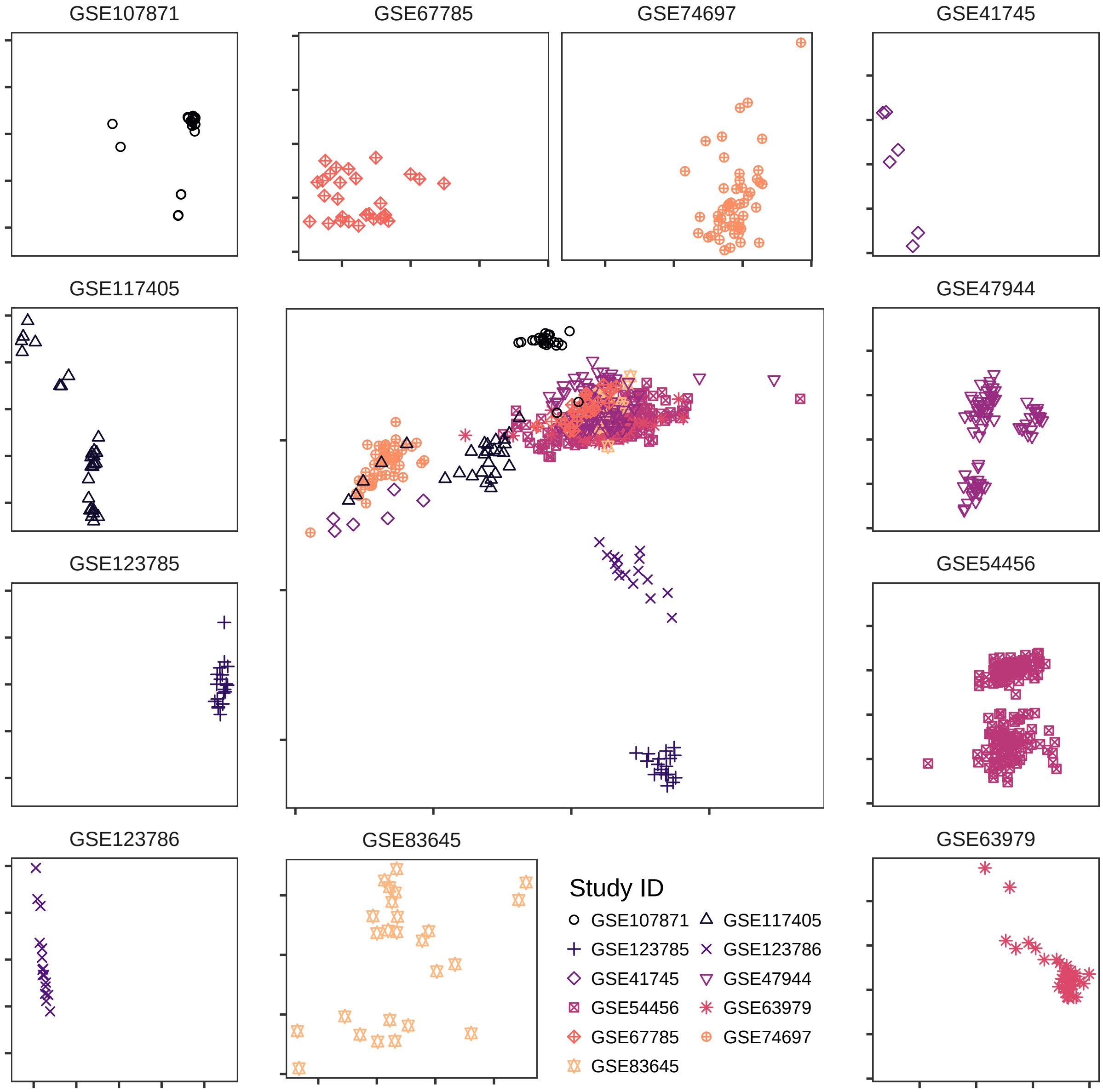

Figure 5: Comparison of the centralized and local PCA. The central plot shows the centralized PCA with all data combined, computed for instance with `SUB-IT`. The smaller plots show each PCA computed only using the local data. The individual plots do not allow to obtain an overview of the data in the same manner than federated PCA. Furthermore, apparent outliers such as in GSE107871 may not be outliers in the larger analysis. Two data points of this data set that look like outliers cluster with the bulk of the data in the combined analysis.

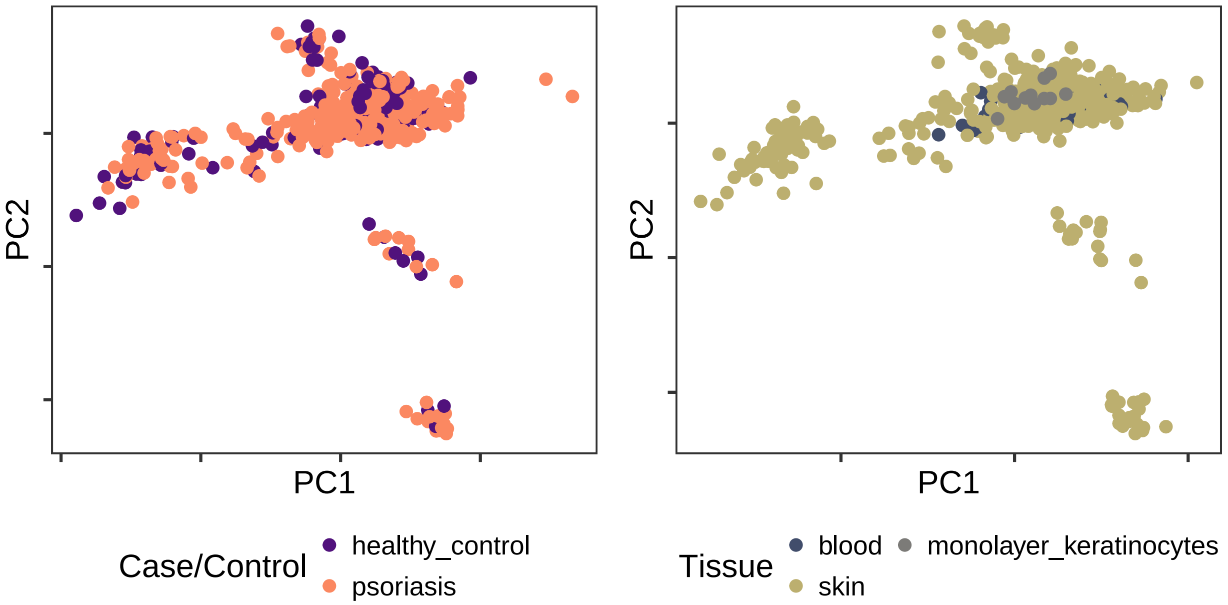

Figure 6: Further sample visualization with respect to other covariates. For further downstream clustering, the batch effect (see fig. 5) would have to be removed. Differential Gene expression tools such as Flimma could be used to find differentially expressed genes in a federated fashion. The model employed in Flimma [11] accounts for batch effects. In this case, the PCA could be used for sample selection. For this figure, instead of using the original data projections, the mean and covariance matrix of the projected data was computed. Then, using a multivariate Gaussian distribution, artificial data points were created to visualize the samples at the aggregator. This way, the analysis is more private.

2.1 Practical implementation – additional run times

Data set	Algorithm	Sites	Time[s]	Iter.	MB
random	P-COV	5	3.1	1	12
		3	3.4	1	6
	AP-STACK	5	2.8	1	16
		3	3.3	1	8
	QR-PCA	5	2.8	1	11
		3	4	1	5
	SUB-IT	5	778	465	6735
		3	492	439	2966

Table 1: Results of the federated test runs using randomly generated data. Empirical runtimes of the PCA algorithms for different data sets averaged over 5 runs. `AP-STACK`, `P-COV` and `QR-PCA` have low execution times in the order of seconds. The low number of executions does not allow to rank the algorithms further. `SUB-IT` has longer execution times and requires more data transmission.

3 Practical utility study

3.1 Data

For the practical application case, we use two publicly available single-cell data sets, a PBMC dataset from 10X Genomics (`PBMC`), and a myeloid progenitor data set [6] (`PAUL`). The data sets consist of 2638 cells $\times$ 1838 genes and 2730 cells $\times$ 3451 genes respectively. After preprocessing according to standard protocols as presented in the scanpy vignettes [2, 3], the single cell measurements are split horizontally into 5 batches which simulate the sites. The preprocessing of the vignettes is not replicated in a federated fashion, however it could be reproduced as it relies mostly on summary statistics such as mean and variance which can be computed easily in a federated fashion. This arbitrary split disregards potential batch effects related to the experimental protocol, as all the cells stem from the same experiment, but is sufficient to illustrate important pitfalls and challenges of federated machine learning in general.

3.2 Setup

In order to put the simulation results into perspective, we choose three popular single-cell RNASeq applications [1], namely data visualization, data clustering and gene importance scoring as application studies. To illustrate the use of federated PCA in these use cases, we implement standard workflows[2, 3] using regular PCA and using simulated federated code for approximate PCA on the two single cell data sets (`PAUL`, `PBMC`). We follow the same steps as the vignettes. We set $k = 50$, the suggested default, resulting in 100 transmitted intermediate dimensions, when using a factor $k' = 2$. Here, we only compare the results of the approximate federated PCA algorithms since the exact methods do not differ from the centralized case at the expense of computational and communication costs. However, due to the lower number of communication rounds and reduced amount of communicated data, the approximate methods speed up the analysis significantly. Therefore, we investigate whether the results of approximate PCA would be acceptable in practice.Many standard single cell analysis pipelines rely on UMAP [5] as a low dimensional representation of the data. Often, the UMAP is computed on the projections of the data onto the first few eigenvectors, in an attempt to overcome the curse of dimensionality. Since we use the projections of the data, federated PCA methods which lead to a small subspace reconstruction error may be sufficient to reproduce the conclusions of the centralized protocol even when the angles of the eigenvectors differ considerably from the centralized version. As an additional downstream analysis relying on the projected data, we include a reproduction of clustering of the `PAUL` data set results using the Leiden clustering [7] and the PAGA [9] algorithm. In order to identify

correlated and important genes, the eigenvectors themselves (loadings) can be analyzed. For example, the importance of the genes can be scored using the most extreme positive or negative entries of the eigenvector.

3.3 Results

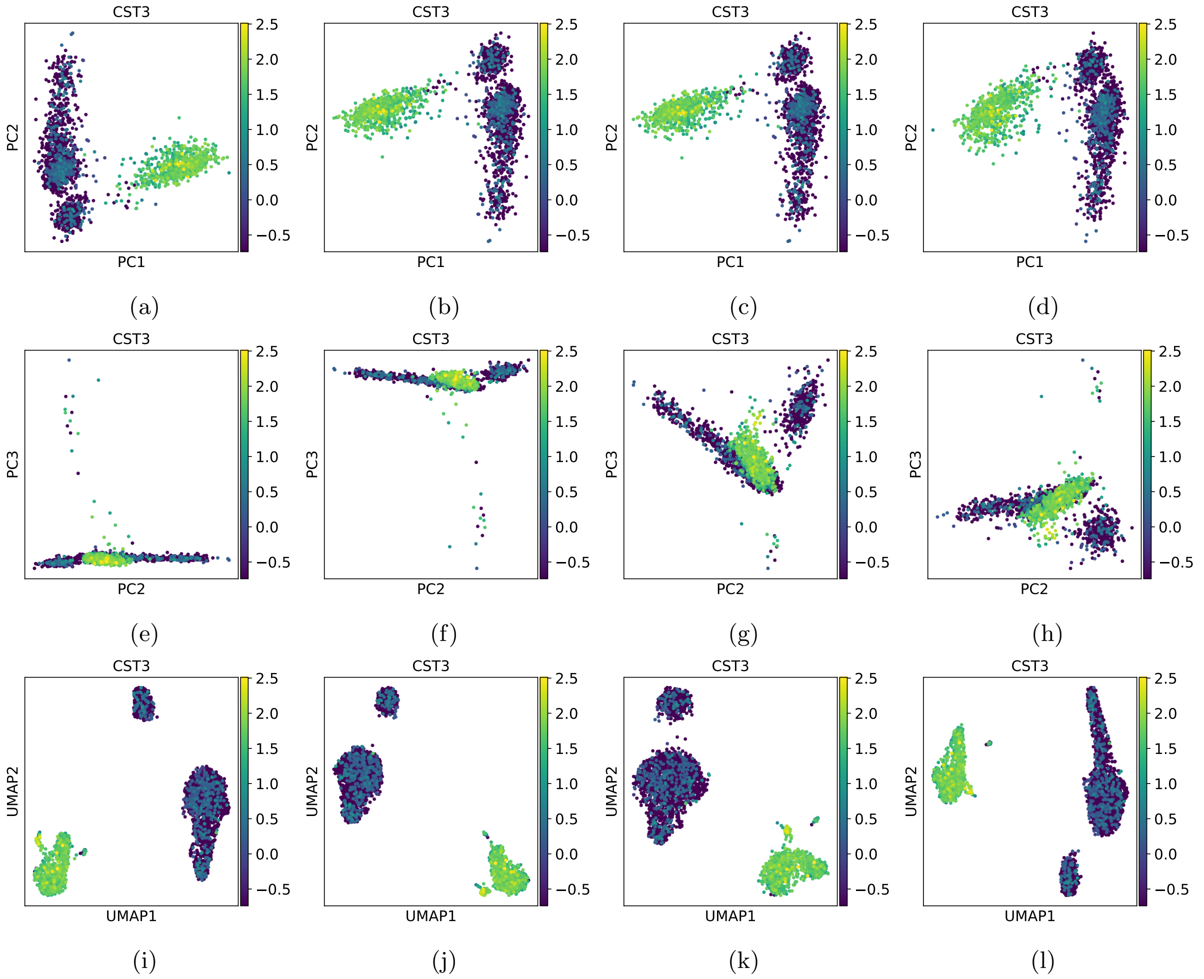

Figure 7: Example application of PCA for single cell RNASeq analysis with different data distributions. a-d) PC1 vs. PC2; e-h) P2 vs. P3; i-l) UMAP1 vs. UMAP2; a,e&i) centralised PCA (baseline); b,f&j) Approximate federated PCA with favorable data/outlier distribution; c,g&k) approximate PCA with unfavorable outlier distribution (one site did not contain outliers, removing an axis of variation); d,h&l) approximated PCA with data split according to the Leiden clustering of data, see supplementary fig. 8. Most visual representations are quite faithful, disregarding the flip of the eigenvectors. Subfigure (g) deviates from the centralized baseline, and the angle between the corresponding eigenvectors is high (see also 11 (a)).

UMAP visualization In fig. 7 the results of approximate federated PCA and the original results for the PBMC data are shown side by side with different data configurations. The first column shows the baseline PC1 vs. PC2; PC2 vs. PC3; and UMAP1 vs. UMAP2 plots, the second and third columns show the same plots for different randomized data splits via different random seeds. The last column contains the figures generated when splitting the data such that each of the three clusters identified by Leiden clustering of the original UMAP embedded space represents a data site (sup. fig. 8). There is not a large visual difference for the plots showing the first two PCs (up to a rotation) for any of the configurations. For the PC2 vs. PC3 plots (2nd row), however, the embedding fig. 7 (g) looks different. Table 11a shows the associated angles between the two randomized splits, corresponding to columns 2 and 3, and the baseline, which reflect a quite

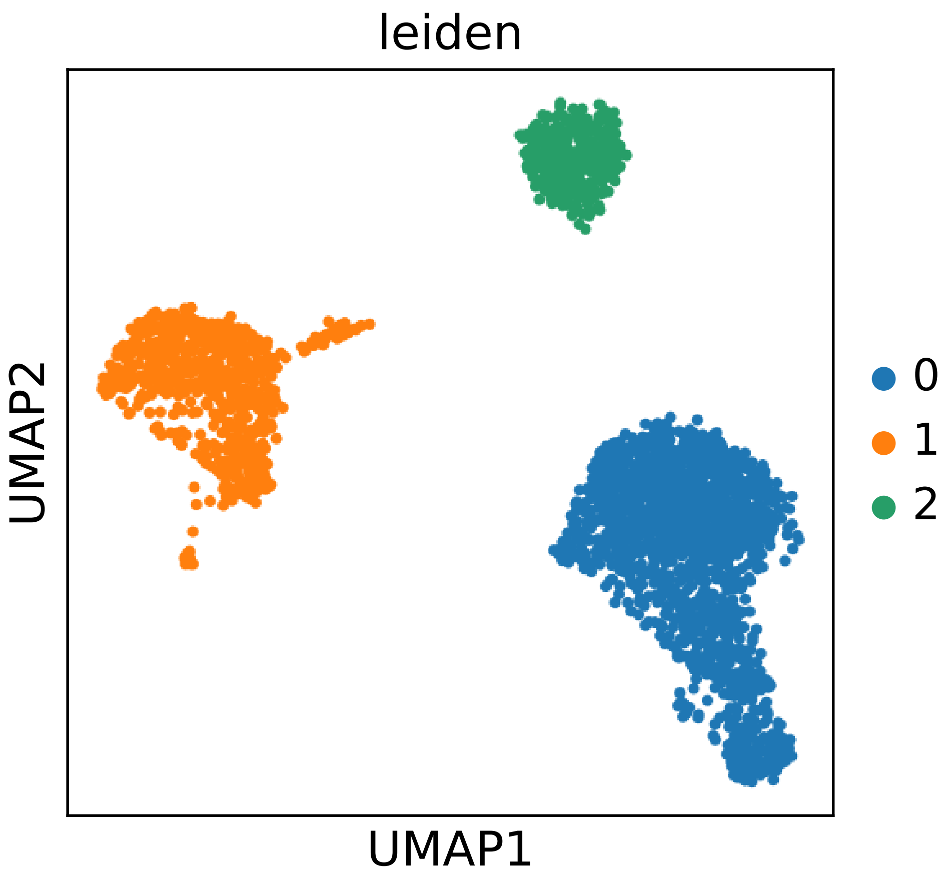

Figure 8: Leiden groups with 3 clusters, using resolution 0.1 to obtain a coarse clustering of the PBMC data. This was done with the centralized workflow. This clustering was then used to create three artificial data sites, each receiving one cluster.

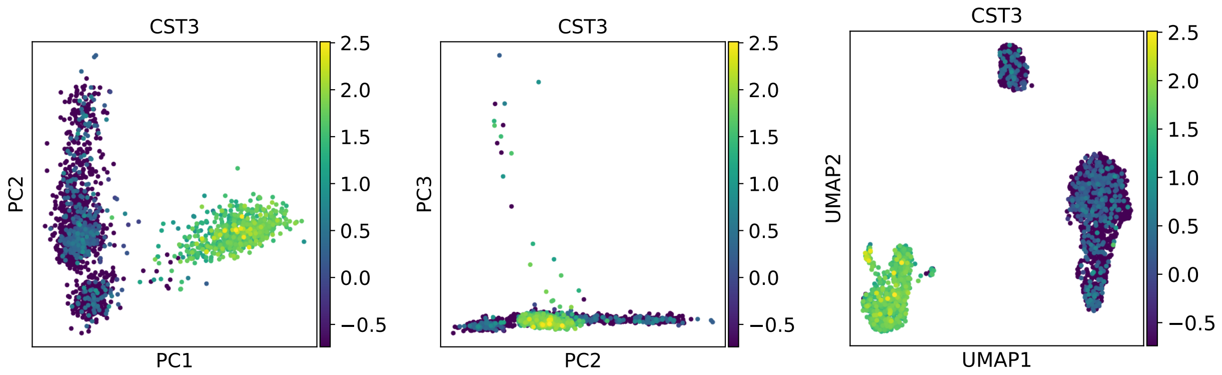

(a) Exact PCA

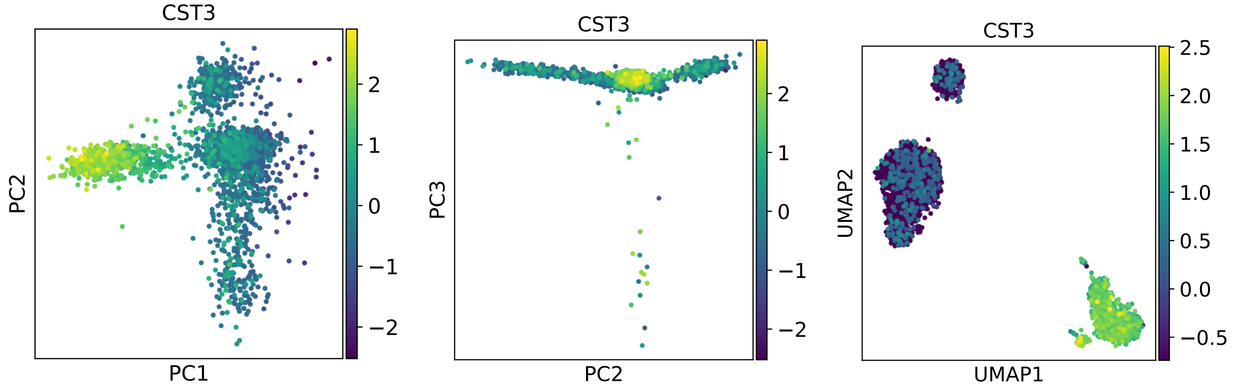

(b) Approximate PCA (k=10)

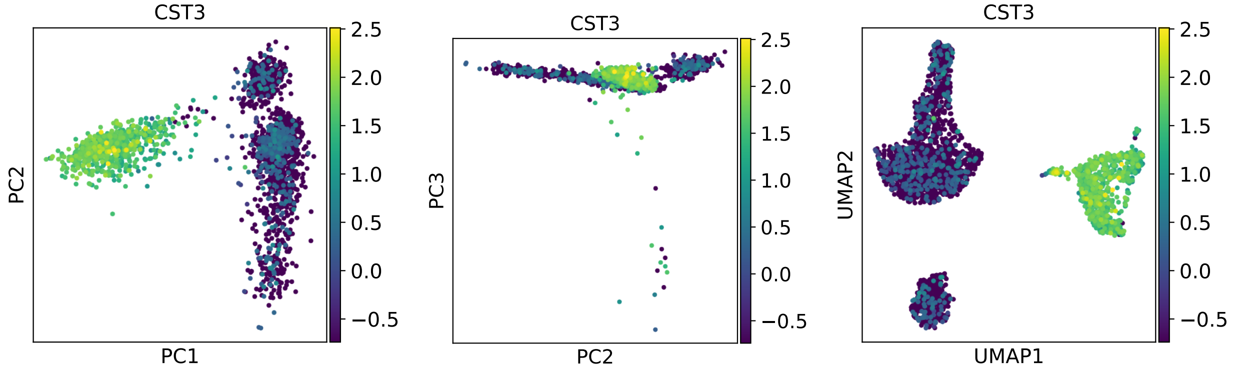

(c) Approximate PCA (k=5)

Figure 9: Example application of PCA for single cell RNASeq analysis using the PBMC data set: UMAP visualizations a) reproduced from the vignette and b) reproduced using approximate federated PCA with k=10 and c) k=5 respectively. Shown are the first vs. second and second vs. third PCs plotted against each other, as well as the UMAP plot for the first two dimensions. The cluster structure is maintained even with lower dimensional approximations of the data. For UMAP visualisation purposes, only few eigenvectors/projections need to be transmitted which is potentially more privacy preserving.

strong deviation of the eigenvectors from the centralized baseline for the latter. The associated UMAP plot (fig. 7 (k)) preserves the three clusters apparent in the original plot.

Upon close investigation, the difference between the eigenvectors using different seeds stem from an unequal distribution of the outliers, for instance the outliers identifiable visually on the third principal axis. These data points create an axis of high variablity, but considering the bulk of the data they may represent erroneous measurements or cells that should be excluded (e.g. dying cells). In the tests, we created arbitrary data partitions, which included those outliers. If the outliers are distributed equally, meaning every artificial site contains some outliers, then the results remain similar to the centralized solution, because the axes of variation in the data are retained. If there are sites which do not have these outliers, this axis of variation is removed from the data for some of the artificial clients and the results are distorted with respect to the centralized solution. In figure fig. 7 (g) the result deteriorates with respect to the centralized solution. A similar result is presented in subfigure (h) where the data is split according to the clustering.

In fig. 9 we include additional figures using a lower k showing essentially the same low dimensional embeddings. This means, the representation can be reproduced using a lower number of eigenvectors. This is favorable for the transmission cost and the privacy.

Downstream analysis – clustering As a downstream analysis relying on the projected data, we include a reproduction of clusterings from the vignettes using the Leiden clustering [7] and the PAGA [9] algorithm. These results are visualized in fig. 10. Using approximate PCA, the pipeline identifies 2 states more than with canonical PCA. The layout of the diffusion maps and graphs are different. While the general structure of the graph seems to be preserved, the upper module of the diffusion map may be prone to different interpretation.

In order to quantify the likeness of the clusterings we compute the macro F1 score, and cluster specific precision and recall. The procedure is as following: first the cluster labels are matched using a global contingency matrix. Labels as matched if they have the highest number of points in common. Then for every cluster precision and recall are calculated:

$$P = \frac{TP}{TP + FP} \tag{1}$$

$$R = \frac{TP}{TP + FN} \tag{2}$$

$$F1 = \sum \frac{2 \cdot P \cdot R}{P + R} * \frac{1}{\#Samples} \tag{3}$$

The macro F1 score is 0.47, cluster wise precision and recall are shown in table 2. Generally, there is some overlap between the clusters but the overall results differ quite strongly from the centralized original solution. We deliverately chose the standard settings, it may be possible to obtain better results which are closer to the original analysis using other parameters, however, we conclude that even for downstream analyses, other than visualization of the first few PCs, approximate PCA is unsuited.

Analysis of loadings In figure 11 we show the cardinality of the overlap of the set of genes identified by using the top 20 largest positive and negative coordinates of the eigenvectors and the associated angle between the eigenvectors for two different random data partitions of the `PBMC` data. While small changes in the angle only lead to minor changes in the gene set, high deviations in the angles also lead to different sets of genes identified. This process is especially vulnerable to outliers which is backed by the observation that the overlap between the baseline genes and the genes identified with approximate PCA is lower for the more distorted eigenvectors where the 'outliers' are distributed unfavorably.

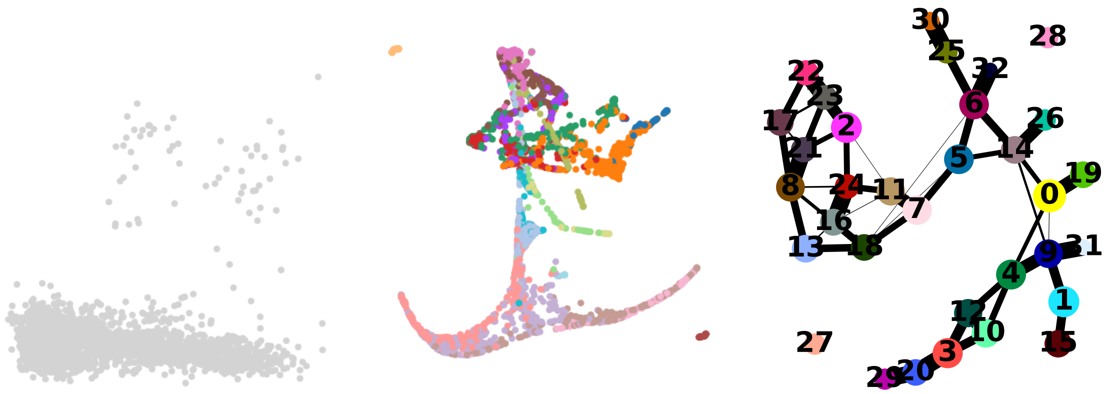

(a) Exact PCA

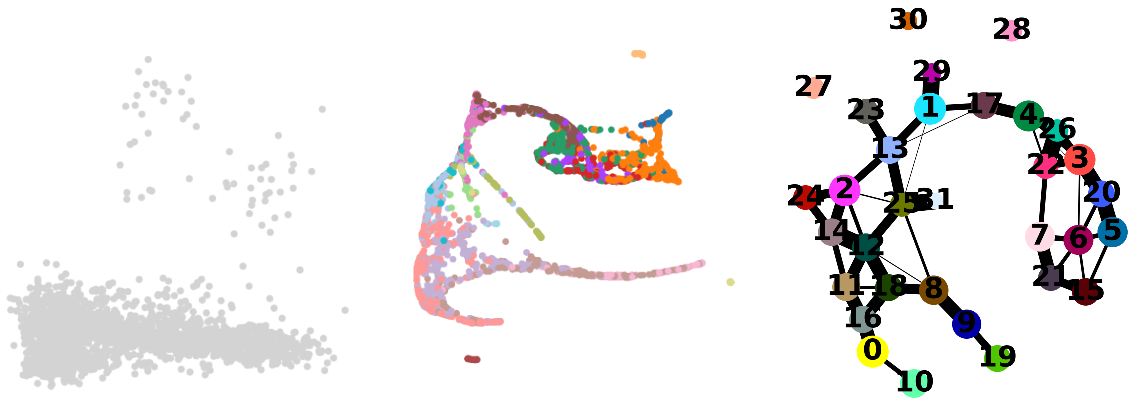

(b) Approximate PCA

Figure 10: Example application of PCA for single cell RNASeq analysis of the `PAUL` data set: Denoised Leiden clustering and PAGA trajectories a) adapted from the vignette and b) reproduced using approximate federated PCA. Table 2 contains quantitative measures on the reproduced figures. Although they look similar, many clusters are not identical.

Cluster	precision	recall	Cluster	precision	recall
0	0.68	0.54	17	0.88	0.72
1	0.71	0.53	18	0.61	0.46
2	0.88	0.79	19	0.81	0.55
3	0.54	0.68	20	0.00	0.00
4	0.57	0.35	21	0.46	0.90
5	0.00	0.00	22	0.00	0.00
6	0.48	0.64	23	0.29	0.44
7	0.45	0.54	24	0.41	0.55
8	0.60	0.66	25	0.00	0.00
9	0.51	0.55	26	0.00	0.00
10	0.70	0.62	27	1.00	1.00
11	0.00	0.00	28	1.00	1.00
12	0.54	0.44	29	0.28	0.97
13	0.00	0.00	30	0.26	1.00
14	0.59	0.55	31	1.00	1.00
15	0.79	0.69	32	0.13	1.00
16	0.35	0.65			

Table 2: Clusterwise precision and recall comparing clustering results of exact and approximate clusterings. The macro F1 score is 0.47. The cluster labels are the labels from the approximate clustering because it yielded more clusters.

1	1.11	1.61
2	2.18	2.21
3	18.15	74.76
4	17.76	74.74
5	8.63	8.34
6	11.48	11.79
7	17.48	19.39
8	26.61	22.32
9	31.92	47.34
10	40.82	36.87
	PBMC	PMBC.1

(a) Angles

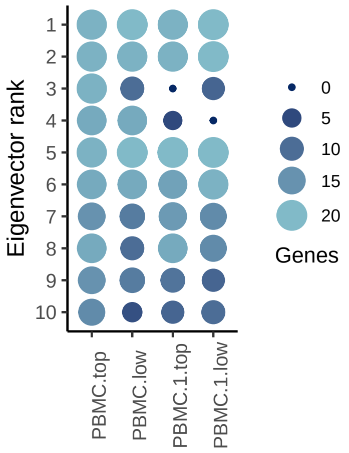

(b) Overlap

Figure 11: Single cell examples: 11a) Angles between the leading centralized and approximate eigenvectors for the `PBMC` dataset using different random seeds (PMBC and PBMC.1), where PBMC.1 has unequally distributed outliers, where some sites did not recieve any. Especially the 3rd and 4th eigenvector have high divergence. 11b Overlap between the genes with the top 20 most positive and most negative loadings identified using approximate and exact PCA. The higher the angle between the eigenvector the lower the concordance of the identified gene sets.

References

[1] Kevin Blighe and Aaron Lun. Pcatools: everything principal component analysis. https://bioconductor.org/packages/release/bioc/vignettes/PCAtools/inst/doc/PCAtools.html#a-loadings-plot, 2021.

[2] Scanpy Documentation. Preprocessing and clustering 3k pbmcs. https://scanpy-tutorials.readthedocs.io/en/latest/pbmc3k.html, 2021.

[3] Scanpy Documentation. Trajectory inference for hematopoiesis in mouse. https://scanpy-tutorials.readthedocs.io/en/latest/paga-paul15.html, 2021.

[4] Lingjuan Lyu, Han Yu, Jun Zhao, and Qiang Yang. Threats to federated learning. *Lecture Notes in Computer Science (including subseries Lecture Notes in Artificial Intelligence and Lecture Notes in Bioinformatics)*, 12500 LNCS:3–16, 2020.

[5] Leland Mcinnes, John Healy, and James Melville. UMAP : Uniform Manifold Approximation and Projection for Dimension Reduction arXiv : 1802 . 03426v2 [stat . ML] 6 Dec 2018. 2018.

[6] Franziska Paul, Ya'Ara Arkin, Amir Giladi, Diego Adhemar Jaitin, Ephraim Kenigsberg, Hadas Keren-Shaul, Deborah Winter, David Lara-Astiaso, Meital Gury, Assaf Weiner, Eyal David, Nadav Cohen, Felicia Kathrine Bratt Lauridsen, Simon Haas, Andreas Schlitzer, Alexander Mildner, Florent Ginhoux, Steffen Jung, Andreas Trumpp, Bo Torben Porse, Amos Tanay, and Ido Amit. Transcriptional Heterogeneity and Lineage Commitment in Myeloid Progenitors. *Cell*, 163(7):1663–1677, 2015.

[7] V. A. Traag, L. Waltman, and N. J. van Eck. From Louvain to Leiden: guaranteeing well-connected communities. *Scientific Reports*, 9(1):1–12, 2019.

[8] N. Vaswani, T. Bouwmans, S. Javed, and P. Narayanamurthy. Robust subspace learning: Robust pca, robust subspace tracking, and robust subspace recovery. *IEEE Signal Processing Magazine*, 35(4):32–55, 2018.

[9] F. Alexander Wolf, Fiona K. Hamey, Mireya Plass, Jordi Solana, Joakim S. Dahlin, Berthold Göttgens, Nikolaus Rajewsky, Lukas Simon, and Fabian J. Theis. PAGA: graph abstraction reconciles clustering with trajectory inference through a topology preserving map of single cells. *Genome Biology*, 20(1):1–9, 2019.

[10] Luke Zappia, Belinda Phipson, and Alicia Oshlack. Splatter: simulation of single-cell rna sequencing data. *Genome Biology*, 2017.

[11] Olga Zolotareva, Reza Nasirigerdeh, Julian Matschinske, Reihaneh Torkzadehmahani, Mohammad Bakhtiari, Tobias Frisch, Julian Späth, David B. Blumenthal, Amir Abbasinejad, Paolo Tieri, Georgios Kaissis, Daniel Rückert, Nina K. Wenke, Markus List, and Jan Baumbach. Flimma: a federated and privacy-aware tool for differential gene expression analysis. *Genome Biology*, 22(1):338, dec 2021.
